# P099: High compliance alcohol based hand rub skin tolerability: alcohol type and application frequency assessment

**DOI:** 10.1186/2047-2994-2-S1-P99

**Published:** 2013-06-20

**Authors:** T Cartner, AV Rawlings, A Saud, MS Bailey, PL McOsker, BT Carr

**Affiliations:** 1 GOJO Industries, Inc., Akron, OH, USA; 2 AVR Consulting Ltd, Cheshire, UK; 3 North Cliff Consultants, Inc., Cincinnati, OH, USA; 4 Carr Consulting, Wilmette, IL, USA

## Introduction

As high Hand Hygiene Compliance (HHC) and Alcohol-Based Hand Rub (ABHR) together play a significant role to reduce the threat of infections, Healthcare Workers (HCW) may use ABHR in excess of 120 times per shift. Little is known regarding how alcohol type or high HHC levels might exacerbate HCW skin condition. This study compares alcohol type and application frequencies on skin at levels representative of high HHC for HCWs.

## Methods

Panelists representing a HCW demographic were studied over a two week period. Following a washout period, skin assessments and treatments were conducted at regular intervals. Three ABHR systems containing 70% alcohol (ethanol, isopropanol, or n-propanol), water, and humectant were used in addition to a control (water and humectant only). Panelist forearms received eight randomized regimens: three alcohol systems applied 20 times per day (standard frequency; SF); three alcohol systems applied 100 times per day (high frequency HF); an untreated skin control; and the water/ humectant system applied 100 times per day. Panelist’s forearms were also washed six times per day at scheduled intervals of a minimal HCW daily routine. Skin redness and dryness, skin hydration, and skin barrier were measured to assess each unique regimen. Analysis of variance was used to assess the individual and interactive effects of alcohol type and application rate, and to compare to the untreated and alcohol-free treatments.

## Results

Compared to the control, elevations in TEWL were observed on days 5 and 8 for the HF-isopropanol regimens. Equally, increased TEWL was observed for the HF-isopropanol regimen compared with the SF & HF-ethanol regimen. Both types of alcohol formulations when applied at HF reduced skin hydration although greater for the isopropanol formulation.

## Conclusion

Both type of alcohol used and the frequency of ABHR application has measureable influence on skin. As the choice of alcohol used in an ABHR has consequences, healthcare facilities moving to or currently at high HHC levels should take this into consideration when selecting an ABHR.

## Disclosure of interest

None declared

